# Analysis of “2·28” KEEPER Chemical Industries Hazardous Chemical Explosion Accident Based on FTA and HFACS

**DOI:** 10.3390/ijerph15102151

**Published:** 2018-09-30

**Authors:** Wei Jiang, Wei Han

**Affiliations:** College of Resources & Safety Engineering, China University of Mining & Technology, Beijing 100083, China; hanwei2324@126.com

**Keywords:** hazardous chemical, explosion accident, FTA, HFACS, accident analysis, unsafe acts

## Abstract

On 28 February 2012, a guanidine nitrate explosion occurred at HEBEI KEEPER Chemical Industries Co., Ltd., China, resulting in 25 deaths, with 4 missing individuals and 46 injured. In order to explore the causal relationship hidden behind this accident, fault tree analysis (FTA) and the Human Factors Analysis and Classification System (HFACS) were used to systematically analyze the incident. Firstly, FTA was used to analyze the causes of the accident in depth, until all the basic causal events causing the guanidine nitrate explosion were identified, and a fault tree diagram of the guanidine nitrate explosion was drawn. Secondly, for the unsafe acts in the basic causal events, the HFACS model was used to analyze the three levels of factors that lead to unsafe acts, including the preconditions for unsafe acts, unsafe supervision, and organizational influences. Finally, based on the analysis results of FTA and HFACS, a complete logic diagram of the causes of the accident was obtained. The FTA and HFACS accident analysis methods allowed for the identification of human factors and the accident evolution process in the explosion accident and provide a reference for accident investigation.

## 1. Introduction

Of the accidents involving hazardous chemical that have occurred in China in recent years, explosions have caused the most serious damage. Du Xiaoyan et al. conducted a statistical analysis of 958 accidents involving hazardous chemicals that occurred in China from 2011 to 2015. The results showed that there were 290 explosion accidents involving hazardous chemicals, accounting for 30.3% of the total number of accidents. As a result of these accidents there were 707 deaths, accounting for 61% of the total deaths caused by the different types of accidents with hazardous chemicals [1]. This is because explosion accidents with hazardous chemicals often occur instantaneously, and the destructive power is often stronger than for other types of accidents. After an explosion, the on-site personnel often do not have enough time to evacuate, and most explosion accidents are accompanied by a secondary derivative disaster, which can easily cause a large number of casualties. This was especially true in the hazardous chemical explosion accident that occurred in HEBEI KEEPER Chemical Industries Co., Ltd. in 2012, which caused25 deaths, with4 missing individuals and 46 injured. The direct economic loss was 44.59 million yuan [2]. The purpose of selecting and analyzing this accident is to discover the causal relationship hidden behind the accident and provide a reference for investigating such accidents.

Some studies have found that human and organizational factors play significant roles in the occurrence of hazardous chemicals accidents. Human and organizational factors play an important role in the occurrence of accidents involving hazardous chemicals. Nivolianitou et al. analyzed major accidents in the petrochemical industry in the European Major Accident Reporting System (MARS), and found that the proportions of human and organizational factors as causes of accidents increased dramatically [3]. Fu Jing et al. analyzed 10 major chemical industry accidents that occurred in China in 2014 using the behavior safety “2–4 model” and found that nine of them were directly caused by unsafe acts by personnel, while the root cause was management error [4]. Zhao Laijun performed a statistical analysis of 3974 hazardous chemical accidents that occurred in China between 2006 and 2017, finding that direct causes of accidents accounted for 50.8%, and factors relating to equipment, facilities, tools, and accessories accounted for 40.35%. However, these factors are directly or indirectly related to human factors [5]. Zhang He da et al. conducted a statistical analysis of 1632 accidents caused by hazardous chemicals that occurred in China from 2006 to 2010. The results showed that most of the accidents with hazardous chemicals were caused by human errors as a direct cause of accidents. However, the root causes could be traced back to the internal corporate management problems [6]. Therefore, it is necessary to identify human factors and organizational factors in accidents with hazardous chemicals.

Some scholars conducted research on human factors and proposed related models, including the Software, Hardware, Environment, and Liveware (SHEL) model, the Reason model, the Human Factors Analysis and Classification System (HFACS) model, the 2–4 model, etc. [7,8,9,10]. Among them, the HFACS model has been widely used in many industries and has been widely recognized. Dekker pointed out that the HFACS is the most powerful tool for human factor analysis of various types of accidents [11]. At present, the application research of HFACS in the aviation field is divided into two categories. The first category is the direct application of the HFACS for accident human factor analysis [12,13], and the second is the use of HFACS as a means of accident cause analysis, combined with other methods for human factor analysis [14,15]. In addition, HFACS for accidental human factor analysis has applications in the coal, maritime, medical, railways, and other industries [16,17,18,19]. When HFACS is applied to areas other than aviation, the HFACS is usually modified to analyze actual cases. In the field of chemical safety, Gong Yunhua et al. analyzed the explosion accident on the Jilin Petrochemical bi-benzene plant accident using HFACS [20], and Zhou Lin et al. analyzed the fire and explosion accident in the Tianjin port with improved HFACS [21]. The application of HFACS in the chemical industry and other fields is quite valuable. However, the HFACS method relies on the association method and lacks systematic normative guidance. For complex accidents, there are many unsafe acts. It is difficult to analyze the horizontal logical relationship between the causes of an accident, and it is difficult to fully reflect the occurrence mechanisms and evolution process of the accident.

Fault tree analysis (FTA) was developed at the Bell Telephone Institute in the United States and was first proposed by Watson and Mearns in 1961. It has been used in accident analysis in various fields, such as the coal, maritime, construction, and railway industries [22,23,24,25]. In the chemical industry, Mulyana et al. used FTA to analyze the probability of failure of key components of liquefied natural gas (LNG) storage tanks [26], and Li Chenyang et al. analyzed fire and explosion accidents in the methane thermal chlorination process based on FTA and Interpretative Structural Modeling (ISM)methods [27].It was found that FTA can logically analyze the cause of the accident and present the evolution process of the accident, compensating for the defects of the HFACS model.

Based on this, this paper draws on the widely used HFACS model in the aviation field as a basis and also integrates FTA to analyze the hazardous chemical explosion accident at KEEPER Chemical Industries Co., Ltd., in order to better realize the safety management of human factors of hazardous chemical explosion accidents, and provide a reference for accident investigation.

## 2. Discussion on Accident Investigation Model

### 2.1. Fault Tree Analysis (FTA)

Fault tree analysis (FTA) is a common analytical method in safety system engineering. It is used to analyze the cause of accidents and evaluate the risk of accidents [28]. FTA is a deductive method, which starts from the specific accident or fault to be analyzed (the top event) and analyzes the causes of the occurrence layer by layer until all the basic cause events leading to the top event are found, and the logical relations between these events are expressed by a logical diagram.

### 2.2. Human Factors Analysis and Classification System (HFACS)

The HFACS was originally proposed by Wiegmann and Shappell. Based on the Reason model, it defines the dominant and implicit factors that cause accidents in the Reason model and describes four levels of human error: (1) unsafe acts; (2) preconditions for unsafe acts; (3) unsafe supervision; and (4) organizational influences [8,9]. Among them, unsafe acts including errors and violations are the dominant form of failure in the Reason model, and the other three levels correspond to implicit failure in the Reason model.

### 2.3. Combination of FTA and HFACS

According to the FTA theory, we can see that the use of FTA to analyze the cause of the accident is clear and logical. In addition, the FTA has the following characteristics. Firstly, it has greater flexibility, and allows for the analysis of not only the impact of equipment and facilities on accidents, but also analysis of human factors and environmental factors and a description of the path of accidents caused by unsafe acts. Secondly, it can be used to find the direct cause of the accident and present the mechanisms of the accident, but it is difficult to systematically analyze the deep-seated causes of the accident, such as unsafe supervision and organizational influences.

Similarly, according to the HFACS theory, the HFACS model considers various factors of the system. In addition to analyzing the unsafe acts of the person causing the accident, it also analyzes the system causes of accidents and the level of each cause in a very scientific and practical manner. However, there are also some shortcomings. Firstly, for more complicated accidents, more unsafe acts occur. The HFACS model is not conducive to analyzing the horizontal logical relationship between the causes of accidents, and it is difficult to fully reflect the evolution process of accidents. Secondly, the analysis process lacks systematic guidance, relying predominantly on the association method. Many reasoning processes rely on the analysis ability of the investigators, so that the quality of the investigators will determine the results of the investigation, and the data obtained are not reliable.

**Figure 1:**
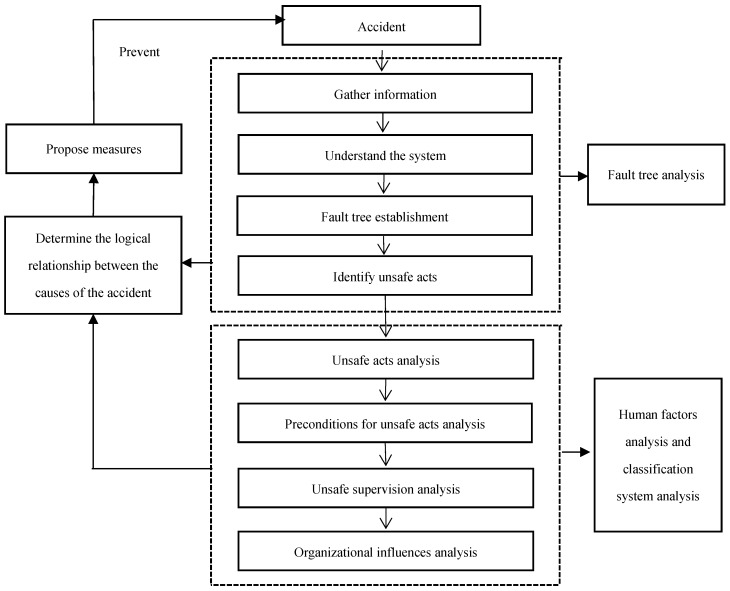
Combination of fault tree analysis (FTA) and the Human Factors Analysis and Classification System (HFACS).

Through the above analysis, it can be seen that the fault tree analysis method allows for finding the horizontal logical relationship between the various causes of the accident, but it is difficult to systematically analyze the deep-seated causes of the accident. The HFACS model is useful for analyzing the deep-seated causes of accidents, but it is not conducive to analyzing the horizontal logical relationship between the causes of accidents. Therefore, this paper combines FTA and HFACS to analyze accidents. First, the accident-related information through multiple channels to understand the system are collected. Then, the FTA theory is used to analyze the accident, and a fault tree map is compiled to find out the unsafe acts in the basic cause events which lead to the top event. Finally, for unsafe acts, the HFACS model is used to analyze the three levels of content that lead to unsafe acts, including the preconditions for unsafe acts, unsafe supervision, and organizational influences. The combination of FTA and HFACS is shown in Figure 1 below.

## 3. Case Study

### 3.1. Accident Description

On 28 February 2012 at 09:04 a.m., a serious hazardous chemical explosion accident occurred at KEEPER Chemical Industries Co., Ltd., located in Shijiazhuang, Hebei province. The explosion occurred at workshop1. The product of workshop 1 was guanidine nitrate, with a designed capacity of 8900 tons/year. The company’s guanidine nitrate production occurred intermittently, and the raw materials for production were ammonium nitrate and dicyandiamide. There were eight reactors in the workshop 1, which were arranged in a single row from north to south and numbered from one to eight.

On the day of the incident, reactors 1–5 were put into use, and reactors 6–8 were deactivated. At about 08:40 a.m. on 28 February, the joint of the heat transfer oil hose at the bottom of reactor 1 was leaking and spontaneously ignited. The workers on duty used the fire extinguisher to extinguish the fire. In the next 20 min, another three to four fires occurred, all of which were extinguished by the workers on duty. At 09:04, reactor 1 suddenly exploded. The high-intensity shock wave generated by the explosion and the high-temperature, high-speed flying metal fragments instantly detonated the guanidine nitrate deposited near reactor 1, causing a secondary explosion. After the accident, workshop 1 was completely destroyed, and the blast formed a pit in the ground on the north side with a length of 14.70 m and a width of 13.50 m. Of the eight reactors, two were crushed, three were blown into two or large pieces, and three reactors were intact. The main structure of workshop 2 on the west side of workshop 1 was seriously damaged, as were the equipment and the pipeline. The west wall of the east side power station was destroyed, and the control panel was damaged seriously. The north side wall was pushed down, and the north side of workshop 6 was also damaged. The glass of the entire factory was shattered. After calculation, the accident explosion equivalent was equivalent to 6.05 tons of Trinitrotoluene (TNT), resulting in 25 deaths, with 4 missing individuals, 46 injured, and direct economic losses of 44.59 million yuan [2].

### 3.2. Fault Tree Analysis

**Figure 2:**
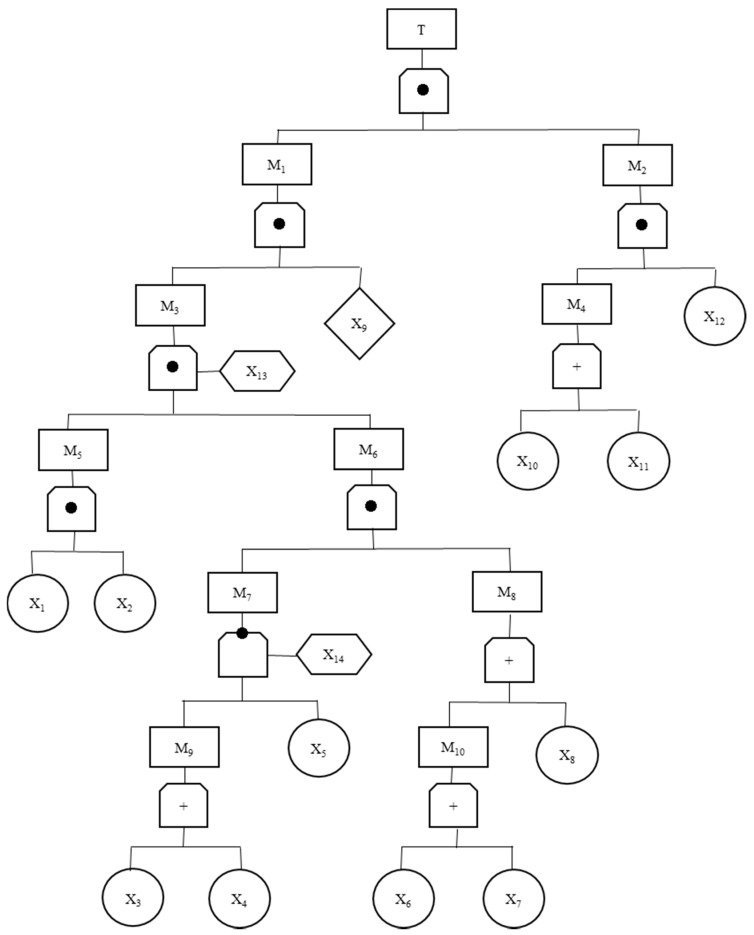
Fault tree diagram of the guanidine nitrate explosion.

According to the accident investigation report, the top event of the accident was identified as a guanidine nitrate explosion. Then, according to the development process of the accident, the reasons leading to the top event and their logical relations were analyzed layer by layer, and the fault tree diagram of the accident was compiled, as shown in Figure 2 below. The meaning of the code in the figure is shown in Table 1 below.

According to the results of the fault tree analysis, there were 12 basic causal events causing the explosion of guanidine nitrate. Among them, there were eight unsafe acts, namely:

X_1_ The outlet temperature of the heat transfer oil heater was increased.
X_3_ Employees did not review the tightness of the heat transfer oil hose connection.
X_6_ The operator removed the temperature indicator in the reactor.
X_7_ Maintenance of important equipment was not performed regularly.
X_8_ The exchange of information between employees and supervisors was not timely.
X_10_ On-the-job operation occurred without effective training.
X_11_ Employees were unclear about hazards such as those related to guanidine nitrate.
X_12_ There was a failure to find the problem in time.

### 3.3. HFACS Analysis

In this accident, there were many unsafe acts. Taking “the outlet temperature of the heat transfer oil heater was increased” as an example, the HFACS model is used to analyze the three levels of content that lead to unsafe acts, including the preconditions for unsafe acts, unsafe supervision, and organizational influences. According to the accident investigation report, the main responsibilities and tasks of the employee, workshop director, supervisor, and manager involved in the accident are shown in Table 2.

#### 3.3.1. Unsafe Acts

The workshop director arbitrarily increased the temperature of the heat transfer oil heater outlet by 40 °C, which was one of the important reasons for the accident. Although this sped up the material melting speed and reaction speed, the temperature of the material in the reactor was close to the deflagration point of guanidine nitrate (270 °C), which presented a major safety hazard in terms of the subsequent explosion of guanidine nitrate and ammonium nitrate. The acts of the workshop director were planned and purposeful, but the decisions were poor. Therefore, this was classified as a “decision error”.

#### 3.3.2. Preconditions for Unsafe Acts

The unsafe act of the workshop director was caused by insufficient training. As the direct manager of the workshop, the workshop director has the right to directly operate all the equipment and facilities in the workshop. At the same time, he should be responsible for the safety of the production of guanidine nitrate in the workshop and lead the workshop staff employees to complete the production plan. However, according to the accident investigation report, the workshop director had only graduated from junior high school and had not received professional and effective training, which caused him to have an insufficient understanding of the hazard information of hazardous chemicals, particularly with respect to the explosion limit, thermal stability, and chemical stability of guanidine nitrate.

Lack of relevant knowledge led the workshop director out of the safe operation range as he increased the temperature of the heat transfer oil heater outlet. It could be seen that the workshop director did not have the ability to correctly implement safe production procedures, meaning that the workshop director had not prepared for the tasks of his position. This was identified as “personal readiness”.

#### 3.3.3. Unsafe Supervision

In terms of “unsafe supervision”, there were two types of human error. According to the accident investigation report, in order to pursue the output and benefit, the company incorrectly implemented the “piece rate system” of the workshop production. This refers to the calculation of wages according to the quantity of qualified products and the pre-specified unit price. It does not directly use labor time to measure labor compensation but uses labor results in a certain period of time to calculate labor compensation. This resulted in a greater capacity for production, which led to the unsafe act of the workshop director to a certain extent. Therefore, this was classified as “planned inappropriate operations”. In addition, the defects of the workshop director in the “personal readiness” implied that the supervisor did not provide appropriate guidance and training for the employees in their daily work. Therefore, this was identified as “inadequate supervision”.

#### 3.3.4. Organizational Influences

The above analysis revealed the defects of KEEPER Chemical Industries Co., Ltd. in “organizational influences”. In terms of “resource management”, this company had insufficient training in human resources. China’s standard “Guidelines for process safety management of petrochemical corporations” (AQ/T 3034–2010) issued in 2010 mentioned that corporations should clearly formulate specific training requirements for each position according to the characteristics of the job and the skills they should possess. In addition, corresponding training plans should be prepared and implemented, and the training plans should be reviewed and practiced regularly to ensure that employees understand the hazards of the process system [29]. The acts of the workshop director and the manager indicated that the national standards were not strictly enforced within the company.

In terms of “organizational climate”, the person in charge of the company simply pursued the output and benefits, and implemented the “piece rate system” of the workshop production; in order to complete the production task, the workshop director increased the temperature of the heat transfer oil heater outlet by 40 °C. KEEPER Chemical Company’s corporate leaders and workshop directors did not prioritize safety problems. They believed that the ultimate goal of production was to maximize benefits and to reflect the safety concept of “light safety, heavy benefit” within the company. It can be seen that the defects in the “organizational climate” were the root cause of the unsafe act of the workshop director.

The HFACS model was used to analyze other unsafe acts in turn, and the analysis results are shown in Table 3 below.

### 3.4. Logical Diagram of Accident Causes

**Figure 3:**
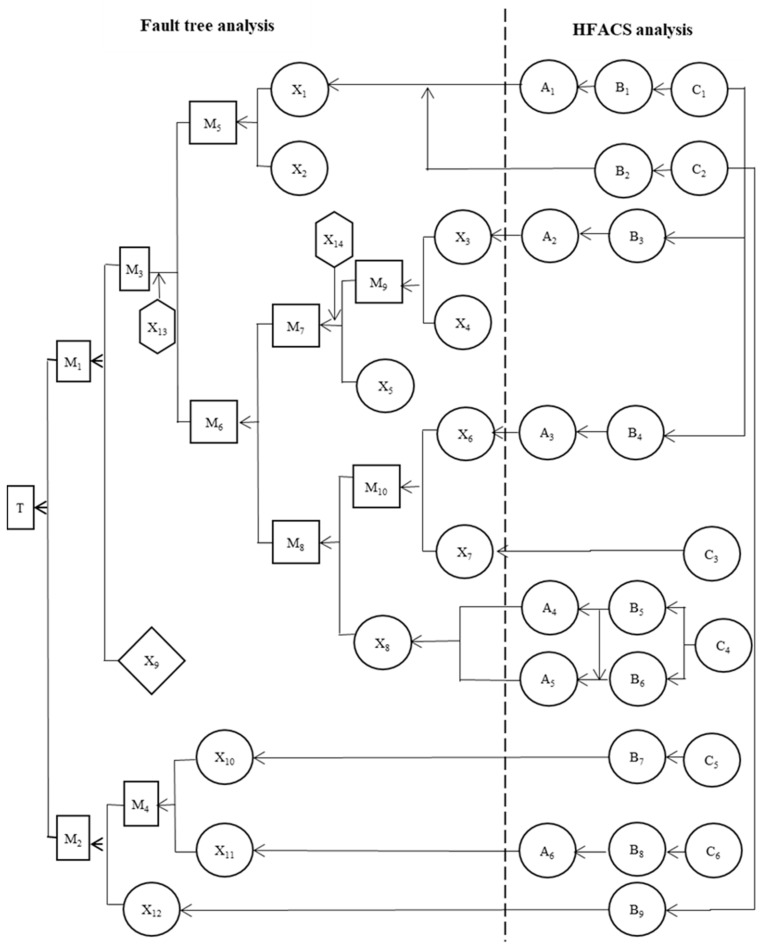
Logical diagram of accident causes.

According to the analysis results of FTA and HFACS, a logical relationship between the causes of accidents was determined, as shown in Figure 3 below.

## 4. Conclusions

Through the research on FTA and HFACS theory, it was found that FTA aided in finding the horizontal logic relationship between various causes of accidents, but it presented difficulties in systematically analyzing the deep-seated causes of accidents. In contrast, the HFACS model was helpful for analyzing the deep-seated causes of accidents, but it was not conducive to analyzing the horizontal logical relationship between the causes of accidents. Therefore, the paper combined FTA and HFACS to analyze accidents. Firstly, accident-related information was collected. Secondly, the FTA theory was used to compile the fault tree diagram, clarify the development process of the accident, and find out the unsafe acts in the basic causal events. Finally, for the unsafe acts, the HFACS model was used to conduct an in-depth analysis of unsafe acts to find the hidden causes of accidents. The feasibility of combining two methods of FTA and HFACS for accident analysis was verified through the investigation of the hazardous chemical explosion accident at KEEPER Chemical Industries Co., Ltd., and several conclusions were obtained.

The use of FTA to present a graphical representation of the occurrence of this explosion accident intuitively and accurately described the complete process of accident development and the logical relationship between the various causes, determining the unsafe acts leading to the accident. According to the definition and classification at all levels of indicators of unsafe acts of HFACS, the unsafe acts that caused the accident were classified into three types: decision errors, skill-based errors, and routine violations.

For the unsafe acts, the HFACS model was used to conduct in-depth analyze the three levels of preconditions, unsafe supervision, and organizational impact of unsafe behavior. The hidden factors that led to the accident are discovered, such as personal readiness defects, inadequate supervision, supervisory violations, insufficient resource management, lack of relevant procedures, and poor safety climate.

According to the analysis results of FTA and HFACS, the logical relationship between the causes of the accident was clarified, and a complete logic diagram of the cause of the accident was drawn.

From the results of the case analysis combined with the FTA and HFACS accident analysis methods, it is possible to more accurately identify the cause of the explosion of hazardous chemicals, and to present the necessary explanations for the details of the accident process. Finally, based on the analysis results, a reference for accident investigation and prevention was provided.

Further research directions are to analyze multiple hazardous chemical explosion accidents based on the method used in this paper, and find common causes leading to hazardous accidents so as to have a deeper understanding of the common accident characteristics of hazardous chemical explosions.

## Figures and Tables

**Table 1:** Code meanings.

Code	Meaning	Code	Meaning
T	Guanidine nitrate explosion	X_3_	Employees did not review the tightness of the heat transfer oil hose connection
M_1_	High-intensity shock wave, high-temperature metal fragments	X_4_	Heat transfer oil hose aging
M_2_	Guanidine nitrate retained near reactor 1	X_5_	The temperature of the pipeline inside the insulation layer wastoo high
M_3_	The guanidine nitrate in reactor 1 exploded	X_6_	The operator removed the temperature indicator in the reactor
M_4_	The produced guanidine nitrate was not transported in time	X_7_	Maintenance of important equipment was not performed regularly
M_5_	The temperature in the reactor was close to the deflagration point of guanidine nitrate	X_8_	The information exchanged between employees and supervisors was not timely
M_6_	The temperature at the bottom of the reactor increased	X_9_	Existing transmission medium
M_7_	The heat transfer oil leaked and spontaneously ignited	X_10_	On-the-job operation without effective training
M_8_	Improper fire emergency disposal	X_11_	Employees wereunclear about hazards such as those relating to guanidine nitrate
M_9_	The heat transfer oil hose connection was not tight	X_12_	Failure to find the problem in time
M_10_	The temperature of the material in the reactor was out of control	X_13_	The temperature in the reactor reached the deflagration point of guanidine nitrate
X_1_	The outlet temperature of the heat transfer oil heater was increased	X_14_	The heat transfer oil ignition point was reached
X_2_	Sound and light alarm, automatic power off device failure	-	-

**Table 2:** The main responsibilities and tasks of each personnel category involved in the accident.

Personnel Category	Main Responsibilities and Tasks
Employee	Works under the leadership of the workshop director, complies with the relevant safety management system of the enterprise
Workshop director	Responsible for the safe production of the workshop, leading the employees to complete the working scheme
Supervisor	Responsible for the company’s safety production and management, assisting the manager in completing the development and implementation of the safety management system, and providing adequate and appropriate supervision, incentive, guidance, and training for employees and workshop director
Manager	Implements national and local laws and regulations, fulfills the responsibility of the first person responsible for production safety, and presides over the development of relevant procedures, documents, and various rules and regulations for safe production

**Table 3:** Human error analysis using HFACS.

Unsafe Acts	Preconditionsfor Unsafe Acts	Unsafe Supervision	Organizational Influences
X_1_ The outlet temperature of the heat transfer oil heater was increased (Decision errors)	A_1_ Workshop director lackedawareness of chemical hazards (Personal readiness)	B_1_ Employees were not provided with adequate training and guidance on hazardous chemicals knowledge (Inadequate supervision) B_2_ Workshop production implemented a “piece rate system” (Planned inappropriate operations)	C_1_ Insufficient human resource management (Resource management) C_2_ Poor safety climate (Organizational climate)
X_3_ The employee did not review the tightness of the heat transfer oil hose connection (Skill-based errors)	A_2_ The employee checked for omissions and lacked skills (Physical/mental limitations)	B_3_ Did not provide adequate guidance on professional skills to employees (Inadequate supervision)	C_1_ Insufficient human resource management (Resource management)
X_6_ The operator removed the temperature indicator in the reactor (Decision errors)	A_3_ The operator was not aware of the importance of the temperature indicator in the reactor (Personal readiness)	B_4_ No training or explanation on equipment and facilities and their functions provided to employees (Inadequate supervision)	C_1_ Insufficient human resource management (Resource management)
X_7_ Maintenance of important equipment was not performed regularly (Routine violations)	-	-	C_3_ Didnot pay attention to the management of important equipment and facilities (Resource management)
X_8_ The information exchanged between employees and supervisors was not timely (Skill-based errors)	A_4_ Insufficient experience and ability to handle complex situations (Physical/mental limitations) A_5_ Lack of teamwork among employees (Communication and coordination)	B_5_ Failure to provide adequate emergency guidance to employees (Inadequate supervision) B_6_ There was no commander at the scene of the fire (Inadequate supervision)	C_4_ No emergency response procedure established (Organizational process)
X_10_ On-the-job operationwithout effective training (Routine violations)	-	B_7_ Failure to implement relevant rules and regulations (Supervisory violations)	C_5_ Defects in the supervision and management mechanism (Organizational process)
X_11_ Employees were unclear about hazards such as those related to guanidine nitrate (Skill-based errors)	A_6_ Workshop employees did not have the ability to identify hazards (Personal readiness)	B_8_ No guidance and training on hazard identification were provided to employees (Inadequate supervision)	C_6_ Hazard source identification procedure not established (Organizational process)
X_12_ Failed to find the problem in time (Skill-based errors)	-	B_9_ Didnot pay attention to the production situation of the workshop and the management of production products (Inadequate supervision)	C_2_ Poor safety climate (Organizational climate)
